# Post-Acute Care for Medicare Beneficiaries in Accountable Care Organizations

**DOI:** 10.1093/geroni/igaa057.061

**Published:** 2020-12-16

**Authors:** Erin Colligan, Brittany Branand

**Affiliations:** NORC at the University of Chicago, Bethesda, Maryland, United States

## Abstract

Post-acute care (PAC) is a component of health-care utilization and spending that is subject to the discretion of providers. Prior research has demonstrated that Accountable Care Organizations (ACOs) recognize PAC as a logical target for increased efficiency and cost savings. As part of the evaluation of the Centers for Medicare & Medicaid Services (CMS) Next Generation ACO (NGACO) Model, we investigated NGACOs’ approaches to PAC services and the impact of these efforts on utilization and cost using a mixed-methods study design. We conducted interviews and surveys with NGACO leadership and providers and performed a difference-in-differences analysis of utilization and spending based on Medicare claims data. We found that NGACOs focused specifically on establishing partnerships with skilled nursing facilities (SNF) to facilitate transitions in care by establishing new channels of communication, sharing performance data, embedding staff in SNFs, and (in some cases) sharing financial risk. We observed a statistically significant decrease in SNF spending, a trend toward fewer SNF days, and statistically significantly lower expenditures for other PAC settings (e.g., inpatient rehabilitation and long-term acute care facilities). These findings suggest that NGACOs have contributed to improving transitions in care and diverting beneficiaries from intensive PAC settings. Nonetheless, the reduction in PAC spending alone did not translate to a decline in total cost of care. Future ACOs may need to expand their focus to the inpatient utilization and spending that precedes PAC in order to impact total cost of care.

